# Draft genomes of 10 skin bacteria isolated from human forearm

**DOI:** 10.1128/mra.00437-26

**Published:** 2026-06-15

**Authors:** Aline Rosin, Maya Sophie Kissner, Lisa Lemoine, Klaus Neuhaus

**Affiliations:** 1 Department of Pesticides Safety, German Federal Institute for Risk Assessment (BfR) 27652, Berlin, Germany; 2 Institute of Biotechnology, Technical University of Berlin 26524, Berlin, Germany; 3 Core Facility Microbiome, ZIEL – Institute for Food & Health, Technical University Munich, Freising, Germany; Wellesley College, Wellesley, Massachusetts, USA

**Keywords:** skin microbiota, skin bacteria, whole-genome sequencing

## Abstract

The human skin microbiome plays a crucial role in skin homeostasis. In this study, we present the draft genomes of 10 bacterial strains isolated from the volar forearms of five healthy donors.

## ANNOUNCEMENT

Skin bacteria constitute the most prominent microorganisms on human skin. They influence skin physiology through various cellular processes and thereby play a crucial role in human health and disease (1, 2). To enable comprehensive characterization of 10 skin bacteria isolated from healthy forearm skin, we conducted whole-genome sequencing.

Skin bacteria were isolated from the volar forearms of five healthy donors (Table 1) in Berlin, Germany (52.53086651°N, 13.29792229°E) using sterile swabs (OmniSwabs, QIAGEN GmbH, Hilden, Germany). The procedure was approved by the German Federal Institute for Risk Assessment (BfR) institutional SFP committee, serving as the internal ethics committee (approval no. 1323-107; data protection internal reference no. BfR-182). No bacterial isolates can be linked to individual donors. Skin swabs were incubated in 1 mL Dulbecco’s Phosphate-Buffered Saline Solution (DPBS; Pan-Biotech, Aidenbach, Germany) at 600 rpm shaking and 32°C for 10 min. The bacterial suspension was plated on Lysogenic Broth (LB; Carl Roth, Karlsruhe, Germany) agar plates and incubated at 32°C until bacterial colonies became visible. Individual colonies were picked and repeatedly streaked onto LB agar plates to obtain pure cultures. For initial phylum level identification, selected bacterial isolates were subjected to Sanger sequencing of the V_1_–V_3_ region of each 16S rRNA gene. DNA was isolated using the Blood and Tissue Kit (QIAGEN GmbH, Hilden, Germany) according to the manufacturer’s instructions. The V_1_–V_3_ region of the 16S rRNA gene was amplified with forward (5′-AGA GTT TGA TCA TGG CTC AG-3′) (3) and reverse (5′-GTA TTA CCG CGG CTG CTG-3′) (4) primers with following settings: 95°C for 5 min; 30 cycles of 95°C for 30 s, 58°C for 40 s, 68°C for 40 s; and a final extension at 68°C for 5 min. Sanger sequencing was performed at Eurofins (Ebersberg, Germany). Sequence reads were identified using “16S-based ID” from ezbiocloud.net (5). In addition, two *Staphylococcus* subsp. were subjected partial *rpoB* gene sequencing using primer 2491F and 3554R as described previously (6). Whole-genome sequencing was performed on 10 strains, belonging to the phyla Bacillota, Actinomycetota, and Pseudomonadota, commonly isolated from human skin. The bacteria were cultivated in liquid LB medium overnight at 32°C and 200 rpm, and the aforementioned kit was used to isolate the DNA. One microgram DNA was randomly fragmented (mean ~350 bp) using a Covaris ultrasonic disruptor. Fragments were end repaired and A-tailed. Sequencing adapters (i.e., P5 and P7) with 8-nt barcodes (i.e., i5 and i7) were ligated, and the fragments were PCR amplified and cleaned. Strain-specific libraries were pooled and sequenced in PE150 mode using a NovaSeq Xplus series 25B. Reads were trimmed for adapters (Trimmomatic v0.39 + galaxy2), assembled to contigs (SPAdes v4.0.0.0). Assembled genomes (i.e., contigs) were checked for their taxonomy using GTDB-Tk v2.6.1 (7) and subsequently annotated within NCBI using PGAP v6.10 (8). For all programs, default parameters were used. All strains were submitted to the Weihenstephan Strain collection (https://ccinfo.wdcm.org/details?regnum=1163) for distribution on request. Table 1 lists all relevant features of the bacterial genomes.

**TABLE 1:** Overview of genomes and features of the 10 skin bacteria

Isolate^*a*^	Species (WGS closest hit)^*b*^	% identity	No. of paired-end reads	GC content (%)	Total size (bp)	No. of contigs	Coverage (×)	N_50_ value	No. of genes for CDS/rRNA (5S, 16S, 23S)/tRNA
WS 5784	*Micrococcus luteus* NCTC 2665(T)	99.64	13,002,516	72.20	2,523,165	72	778	136,799	2,327/1, 2, 1/48
WS 5785	*Micrococcus luteus* NCTC 2665(T)	99.57	10,195,144	72.88	2,462,890	47	625	217,999	2,235/1, 2, 1/48
WS 5786	*Peribacillus simplex* DSM 8801(T)	100	10,090,166	40.35	5,542,062	69	274	437,268	5,342/7, 3, 2/82
WS 5787	*Kocuria rhizophila* TA68(T)	99.93	8,728,284	70.56	2,779,974	30	474	220,770	2,436/0, 1, 5/47
WS 5788	*Staphylococcus capitis* ATCC 27840(T)	100	7,931,896	33.30	2,496,844	31	479	698,297	2,383/2, 2, 2/59
WS 5789	*Staphylococcus warneri* ATCC 27836(T)	100	8,906,176	32.89	2,445,526	21	549	460,336	2,328/1, 2, 2/47
WS 5790	*Staphylococcus condiment* DSM 11674(T)	99.79	12,047,440	34.68	2,639,330	37	689	423,217	2,510/3, 1, 3/37
WS 5791	*Pseudomonas fulva* NBRC 16636(T)	99.93	10,409,426	61.51	5,180,912	74	303	383,570	4,776/1, 2, 1/67
WS 5792	*Acinetobacter radioresistens* DSM 6976(T)	99.86	11,206,708	41.41	3,226,139	110	684	83,494	3,048/0, 3, 6/61
WS 5794	*Staphylococcus* *epidermidis* NCTC 11047(T)	100	8,380,968	32.30	2,473,685	54	511	198,138	2,348/2, 2, 3/55

^*a*^ WS = Weihenstephan Strain collection.

^*b*^ According to closest hit in GTDB‑Tk v2 (7) after WGS.

## Data Availability

All raw files of this project have been deposited in the NCBI sequence read archive (SRA), and the versions described in this paper are the first version. The BioProject accession number is PRJNA1433427, and the BioSample accession numbers are SAMN56364140 (WS 5784), SAMN56364141 (WS 5785), SAMN56364142 (WS 5786), SAMN56364143 (WS 5787), SAMN56364144 (WS 5788), SAMN56364145 (WS 5789), SAMN56364146 (WS 5790), SAMN56364147 (WS 5791), SAMN56364148 (WS 5792), and SAMN56364149 (WS 5794). The sequencing reads of the V1–V3 region of the 16S rRNA gene have been deposited under the following accession numbers: SRR37556610 (WS 5784), SRR37556609 (WS 5785), SRR37556608 (WS 5786), SRR37556607 (WS 5787), SRR37556606 (WS 5788), SRR37556605 (WS 5789), SRR37556604 (WS 5790), SRR37556603 (WS 5791), SRR37556602 (WS 5792), and SRR37556601 (WS 5794). The rpoB gene sequencing reads can be accessed under SRR37528309 (WS 5788) and SRR37528308 (WS 5794). The raw sequencing reads of the genome have been deposited under the accession numbers SRR37502951 (WS 5784), SRR37502950 (WS 5785), SRR37502949 (WS 5786), SRR37502948 (WS 5787), SRR37502947 (WS 5788), SRR37502946 (WS 5789), SRR37502945 (WS 5790), SRR37502944 (WS 5791), SRR37502943 (WS 5792), and SRR37502942 (WS 5794). The annotated genomes can be accessed under following accession numbers: JBWBCQ000000000 (WS 5784), JBWBCR000000000 (WS 5785), JBWBCS000000000 (WS 5786), JBWBCT000000000 (WS 5787), JBWBCU000000000 (WS 5788), JBWBCV000000000 (WS 5789), JBWBCW000000000 (WS 5790), JBWBCX000000000 (WS 5791), JBWBCY000000000 (WS 5792), and JBWBCZ000000000 (WS 5794).
